# Evaluation of Two Non-Invasive Extracorporeal Strategies for Enhancing Protein-Bound Uremic Toxin Removal During Hemodialysis

**DOI:** 10.3390/toxins18100443

**Published:** 2026-10-13

**Authors:** João G. Brás, Fokko P. Wieringa, Babette H. Lentferink, Simon Kootwijk, Albert H. Westra, Leonard J. van Schelven, Mies van Steenbergen, Joachim Jankowski, Marianne C. Verhaar, Karin G. F. Gerritsen, Jeroen C. Vollenbroek

**Affiliations:** 1 Department of Nephrology, University Medical Centre Utrecht, 3584 CX Utrecht, The Netherlandskootwijk.simon@gmail.com (S.K.); m.c.verhaar@umcutrecht.nl (M.C.V.); k.g.f.gerritsen@umcutrecht.nl (K.G.F.G.); 2 IMEC the Netherlands—Health Research, 5656 AE Eindhoven, The Netherlands; 3 European Kidney Health Alliance (EKHA), 1000 Brussels, Belgium; 4 Department of Medical Technology and Clinical Physics, University Medical Centre Utrecht, 3584 CX Utrecht, The Netherlands; 5 Division of Pharmaceutics, Utrecht Institute for Pharmaceutical Sciences, Utrecht University, 3584 CG Utrecht, The Netherlands; 6 Aachen-Maastricht Institute for Cardiorenal Disease (AMICARE), University Hospital RWTH Aachen, 52074 Aachen, Germany; 7 BIOS Lab on a Chip Group, Faculty of EEMCS, University of Twente, 7500 AE Enschede, The Netherlands

**Keywords:** end-stage kidney disease, protein-bound uremic toxins, hemodialysis, elevated temperature, mathematical modeling, electromagnetic fields

## Abstract

Current hemodialysis (HD) methods inefficiently remove protein-bound uremic toxins (PBUTs) due to their high binding affinity to plasma proteins, mainly albumin. This study aimed to investigate electromagnetic (EM) fields and temperature as potential extracorporeal, non-systemic methods to increase HD PBUT clearance. PBUT release was measured in infusible (caprylate-stabilized) albumin solutions spiked with indoxyl sulfate (IS) using an in vitro dialysis setup including a real-time, in-line IS-sensor. No measurable influence on PBUT release was observed during EM field-tests (f = 1–600 MHz, E <= 1 kV/m), but temperature tests (35–40 °C range) showed an absolute increase in IS free fraction of 0.7%/°C. Furthermore, static equilibrium dialysis tests, including IS, p-cresyl sulfate (pCS) and hippuric acid (HA), showed a free fraction increase for all three PBUTs upon a temperature increase from 37 °C to 43 °C (IS 1.30-fold, pCS 1.12-fold, HA 1.15-fold). Mathematical modeling showed a 1.14-fold decrease in plasma IS concentration after 5 weeks of HD, and exploratory hemocompatibility tests did not reveal pronounced changes over 15 heating cycles at 43 °C under static conditions. Extracorporeal temperature elevation is a promising technique to enhance PBUT removal during hemodialysis, which may be easily combined with current HD machines, does not break sterility, and avoids elevating free PBUTs inside the body.

## 1. Introduction

End-stage kidney disease (ESKD) is marked by a life-threatening loss of kidney function, for which kidney replacement therapy (KRT) is needed to survive. Hemodialysis (HD) is the most widely applied KRT, but it provides limited clearance of protein-bound uremic toxins (PBUTs) [1,2,3]. The resulting accumulation of PBUTs contributes to cardiovascular disease and cognitive decline, and accelerates further decrease in residual kidney function [4,5,6]. PBUTs, along with numerous other endogenous and exogenous compounds, bind mainly to Sudlow’s binding sites I and II of human serum albumin (HSA), which is the most abundant plasma protein. In plasma, PBUTs exist at an equilibrium between bound and unbound (free) states (see Table 1). Healthy kidneys remove PBUTs efficiently through active tubular secretion, but due to their high albumin binding PBUTs are only poorly removed by HD, as only the free fraction can pass through the passive dialysis membrane. Several HD modalities have been evaluated for their effectiveness in removing PBUTs when compared to conventional HD, such as post-dilution hemodiafiltration (HDF), expanded HD (HDx) using medium cut-off membranes and extended HD [7]. However, none of these modalities have consistently reduced PBUT concentrations compared to HD in the long term. Therefore, increasing the removal of PBUTs is an important target on the innovation roadmap for KRTs [8].

Several PBUT removal enhancement techniques have been previously investigated, including the use of adsorbents, chemical displacers, and increased plasma ionic strength (via addition of sodium chloride) [7,14]. The present study investigated the application of (A) electromagnetic fields and (B) increasing blood temperature above 37 °C, both applied to the extracorporeal blood circuit. These two approaches, as well as chemical displacers and elevated ionic strength, aim to increase the free (unbound) fraction of PBUTs. For instance, infusion of caprylic acid, a free fatty acid, increased fractional removal of IS and pCS up to 2.5-fold with in vitro HD [15], while infusion of ibuprofen and furosemide achieved a 2.9-fold increase for IS [12]. Despite their efficacy, chemical displacers and elevated ionic strength face important limitations, such as increased procedural complexity, limited cost-effectiveness, and the need for further safety assessment, which have limited their clinical adoption. Hence, PBUT removal methods should not only enhance clearance but also remain cost-effective, biocompatible, and easily combinable with current HD systems without compromising sterility and—most importantly—without increasing systemic levels of unbound PBUTs. In line with these requirements, the proposed approaches aim to selectively increase the free fraction of PBUTs *only within the dialysis filter*, thereby enhancing dialytic removal while minimizing systemic exposure [16,17].

### 1.1. Approach A: Using Electromagnetic (EM) Fields to Enhance PBUT Removal

EM fields have been reported to increase the dissociation of PBUTs from albumin, either by altering the conformation of albumin or by influencing the electrostatic PBUT–albumin interactions [18,19]. The EM fields application between 1 Hz and 1 MHz reduced the binding affinity of norfloxacin (an antibiotic primarily bound to Sudlow’s site I [20]) to bovine serum albumin (BSA) by approximately 6-fold [21]. In another study, frequencies of 125, 180 and 230 MHz changed the UV/Vis absorption of BSA, indicating conformational changes and increased exposure of amino acid residues to its surroundings [19]. Research applied specifically to PBUT–albumin interactions is however still in the early stages. To this effect, Jankowski et al. patented a device that applies high-frequency EM fields to a dialyzer [22]. Frequencies between 1 and 20 MHz were reported to increase the in vitro dialytic removal of phenylacetic acid (PAA) by 1.3-fold and IS by 1.4-fold, while a range of 110–115 MHz increased PAA removal by 1.3-fold (no data on IS reported for this range). Finally, Hsiao et al. demonstrated that low-frequency electric fields (<1 Hz) applied to electrically conductive mixed-matrix membranes (MMMs) increased the free fraction of p-cresol by 12% compared to standard MMMs [23].

### 1.2. Approach B: Elevated Temperature

It is known that the binding of small anionic organic solutes to the Sudlow binding sites of HSA has a reversible temperature dependency, where an increase in temperature results in a decrease in solute binding [24,25,26,27,28]. Existing literature on the temperature dependency of PBUT-HSA binding predominantly describes a range between 4 and 37 °C [25,28,29], reflecting most experimental and physiological conditions. Thus, data on PBUT binding above 37 °C is limited. Between 25 and 37 °C, an absolute increase in free fraction of 0.3%/°C was reported for IS and HA in serum, 0.2%/°C for pCS and IAA, and 0.1%/°C for CMPF [25,28,29]. In the 37–42 °C range, an increase of 0.1%/°C was reported for IS [15]. In addition, changes in the free fraction of a toxin can be estimated from the thermodynamic properties of the binding reaction [24]. From this model, strongly bound PBUTs (e.g., IS, pCS), which are the least removed by HD, are expected to exhibit a larger *relative* release from albumin than weakly bound ones (e.g., HA) for the same binding site and temperature rise, although this may still correspond to a limited absolute free fraction change. Furthermore, the free fraction increase rate is expected to also rise marginally with temperature.

## 2. Results

For both approaches, an in vitro dialysis setup with recirculation on both sides was used (see Section 5.1 and Section 5.2.2). A 40 g/L albumin solution served as plasma surrogate and was spiked with IS prior to the experiment to achieve a total IS concentration of 60 µM. IS concentration was measured in the dialysate solution during application of (A) the EM fields or (B) temperature variation, using a real-time in-line optical sensor.

### 2.1. Approach A: EM Fields

**Figure 1:**
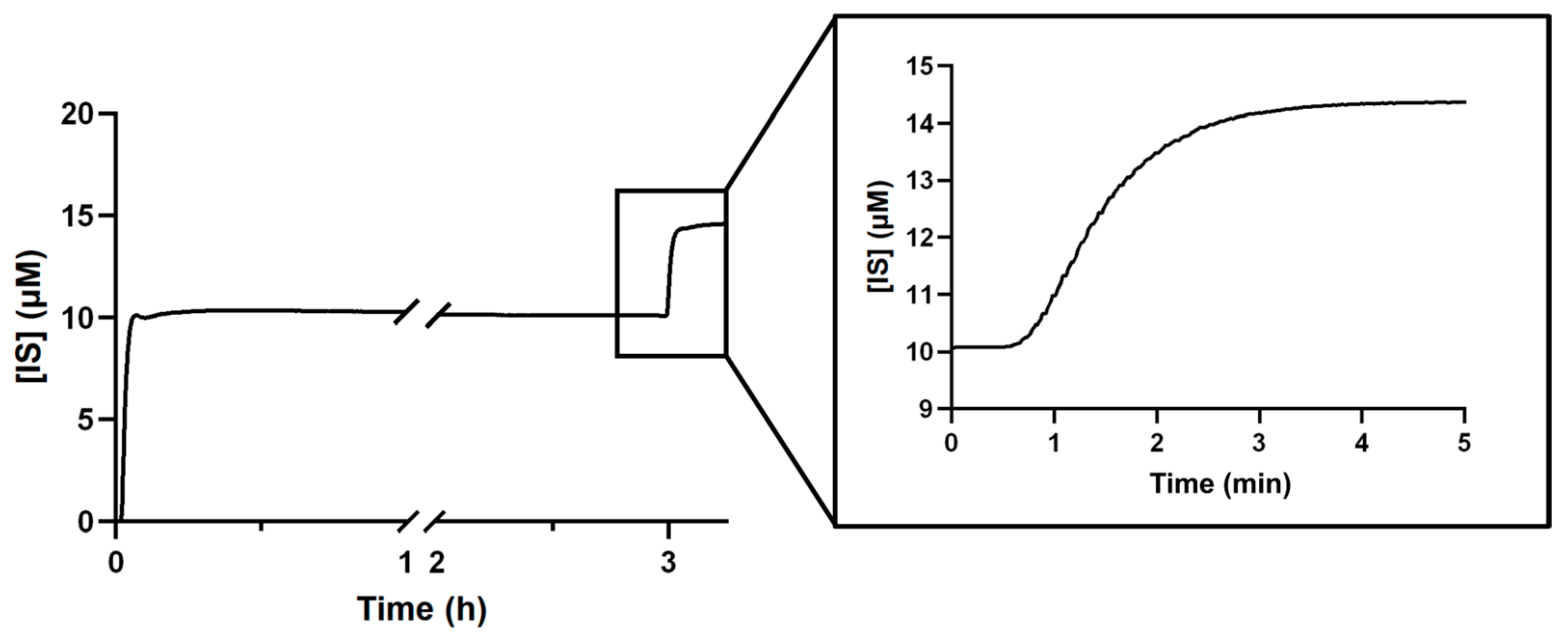
Indoxyl sulfate (IS) concentration in dialysate in response to a step change in IS concentration in the albumin solution without the EM field on.

Figure 1 shows the concentration of IS in the dialysate over time during the experiment. IS concentration at the dialysate side rapidly rises towards an equilibrium level, marking the initial IS concentration. Consequently, from this point in time, any change to the IS concentration in dialysate was assumed to be due to the applied EM field test conditions increasing the free fraction in the albumin side.

First, the time domain response of the optical in-line monitor was checked. After 3 h, the albumin solution reservoir was spiked with additional IS and mixed, aiming for a 50% increase in concentration to simulate an enhanced PBUT release reaction from albumin. This led to the step increase in IS concentration on dialysate visible on the 3 h mark (Figure 1) and a new equilibrium was reached after 4 min, so dwell times at a certain frequency could consequently be kept short.

**Figure 2:**
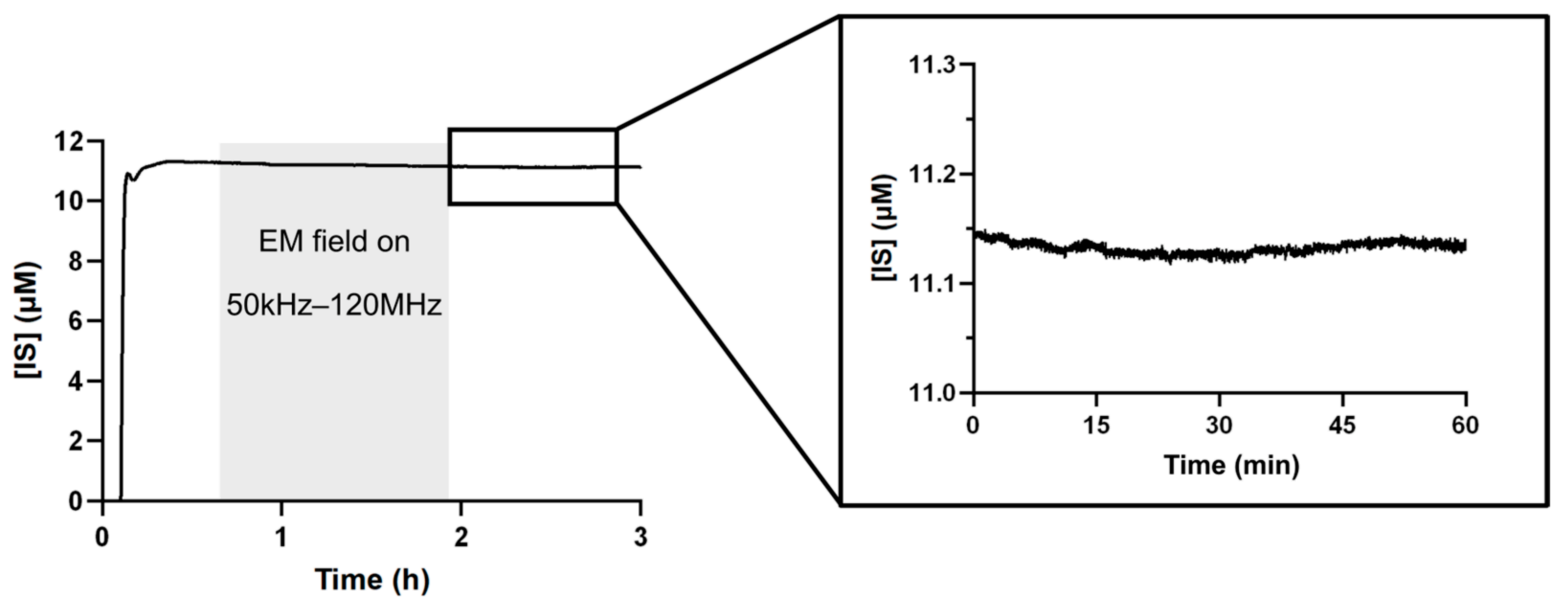
Indoxyl sulfate (IS) concentration equilibrium in dialysate over the course of an experiment. (frequency sweep) f = 50 kHz–120 MHz, dwell time ≈ 22 s, frequency step = 1 MHz, T = 21 °C.

Figure 2 shows the application of EM fields (gray region) with a frequency sweep of 50 kHz to 120 MHz (dwell time at each frequency ≈ 22 s, frequency step = 1 MHz) using the parallel plate setup (see Section 5.1). There was no observed significant change in IS concentration in dialysate with these settings. The zoomed-in section corresponds to an interval without EM exposure and details the sensor’s background noise. No significant changes in IS levels in dialysate were observed from EM field application at any of the tested frequencies (1–600 MHz, see Section 5.1). Included in this range are the frequencies previously reported as most effective by Jankowski et al. [22], which were tested with three electrode arrangements (see Section 5.1 Figure 8). Increasing albumin temperature from room temperature (RT; 21 °C) to 37 °C also did not result in a response to EM fields.

### 2.2. Approach B: Elevated Temperature

#### 2.2.1. Dynamic In Vitro Dialysis

**Figure 3:**
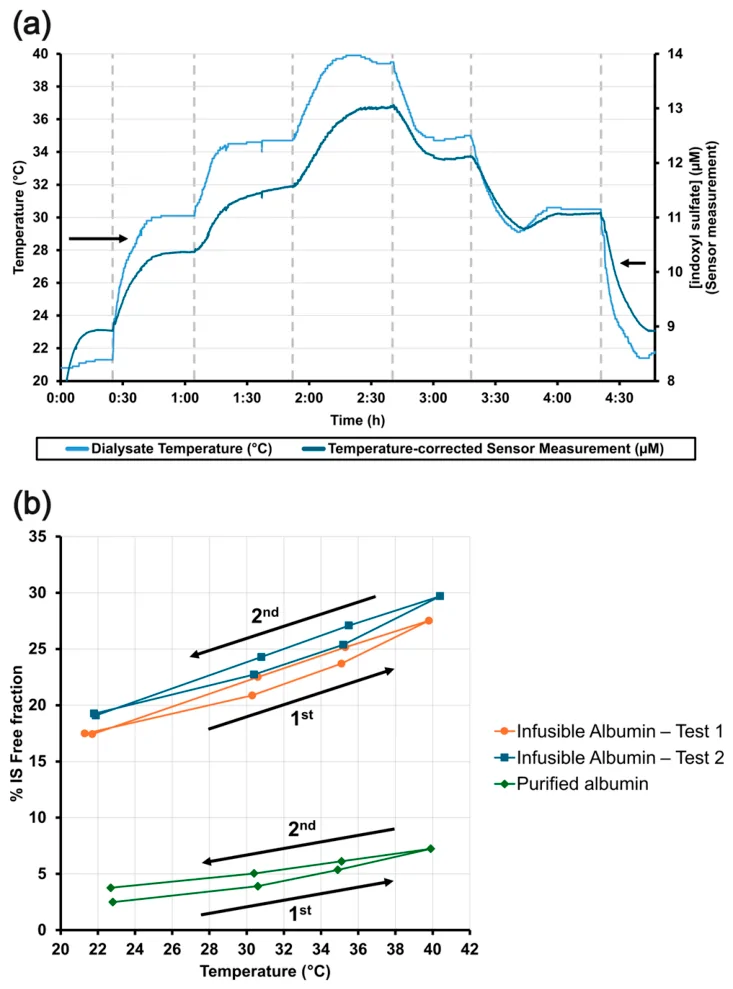
Indoxyl sulfate (IS) dialysate concentration response upon first stepwise increasing and then decreasing temperature (~22, 30, 35, 40 °C and back). (**a**) IS concentration in dialysate (dark blue, right vertical axis) and temperature (light blue, left vertical axis). The dotted vertical lines mark the start of a new temperature step; (**b**) effect of increasing (1st) and decreasing (2nd) temperature on IS free fraction for infusible and purified albumin solutions.

Figure 3a shows the IS free fraction response to first increasing and then decreasing temperature stepwise using an in vitro dialysis setup. Fluid temperature steps resulted in an almost immediate change in IS levels in dialysate, and a stable temperature and IS concentration was reached for each temperature step after ~40 min. This indicates free IS fraction changes with temperature (Figure 3b). Note that the free fraction of IS is higher in the commercial infusible HSA solution when compared to purified albumin (~30% compared to 5–10% at 37 °C). This difference is due to the presence of stabilizers in the infusible solution, particularly sodium caprylate (~4 mM), which is a known Sudlow site II ligand and effective PBUT displacer [15,30]. The infusible solution was used because purified albumin solutions were cost prohibitive with the high required volume. After the peak temperature was reached (~40 °C), the free fraction decreased upon cooling. However, at a given temperature, the values remained higher than those measured during heating until the temperature returned to its initial value, revealing a minor, yet interesting, hysteresis-like behavior.

#### 2.2.2. Rapid Equilibrium Dialysis (RED)

**Figure 4:**
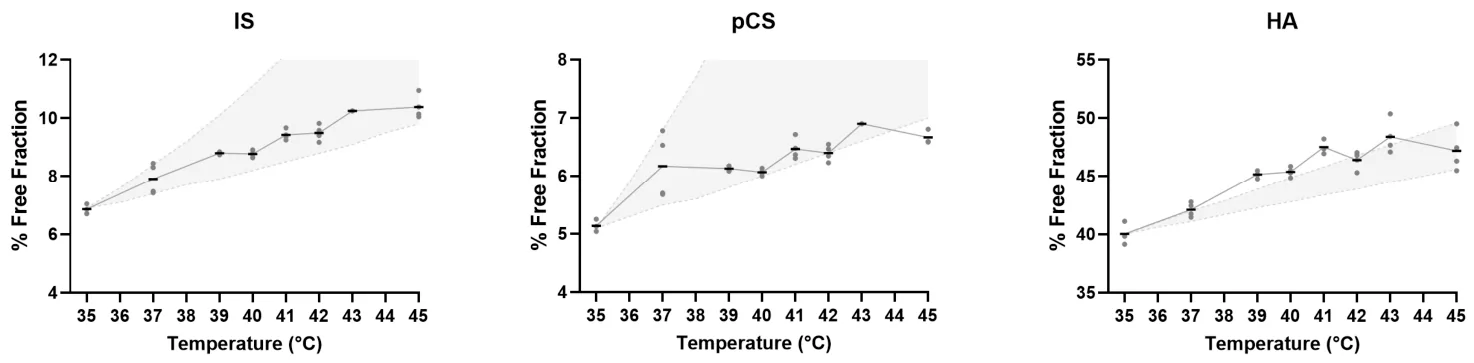
Free fractions of IS, pCS and HA at incubation temperatures between 35 and 45 °C. Data are shown as individual technical replicates, *n* = 1–4, with horizontal lines indicating the mean. The shaded regions represent free fraction intervals resulting from running the model with the highest and lowest reported ΔH° values [35].

Using different experimental and measurement techniques (rapid equilibrium dialysis (RED) and HPLC), the free fractions of IS, pCS and HA were determined at different temperatures (see Figure 4). For all three PBUTs, free fractions increased when elevating temperature from 37 °C to 43 °C (IS 1.30-fold, pCS 1.12-fold, and HA 1.15-fold). The free fractions in Figure 4 are lower than in Figure 3b because the RED tests used purified powdered albumin, which does not contain stabilizers. The shaded region represents the estimated free fraction of PBUTs with increasing temperature, derived using the model described in Section 5.2.1 and using previously published values for free fraction and reaction enthalpy [31,32,33,34]. The determination of enthalpy (∆*H^o^*) values is sensitive to experimental conditions, leading to significant inter-study variability, so free fractions are shown as intervals within the range of reported in vitro ∆*H^o^* values (in kcal/mol: IS, 7.5–20.2; pCS, 6.3–27.5; HA, 4.4–7.6) [35]. The measured values are in accordance with the modeled curve.

#### 2.2.3. Reversibility

**Figure 5:**
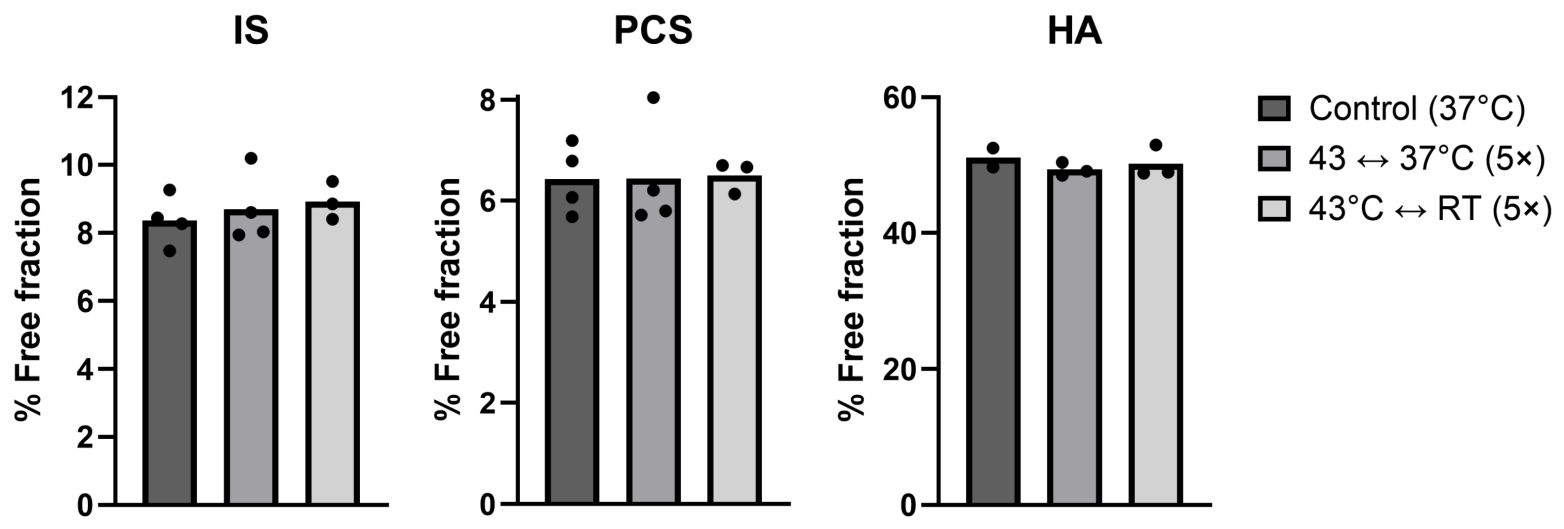
Free fractions of IS, pCS and HA at 37 °C with samples subjected to temperature cycling at different cooling temperatures before incubation. Data are shown as individual technical replicates, *n* = 2–4. RT = room temperature.

To determine whether repeated temperature changes induce persistent alterations in albumin that affect PBUT binding, the PBUT-spiked albumin samples were subjected to temperature cycling before incubation. The PBUT-spiked albumin samples were either kept at 37 °C (control), cycled for five times between 37 °C and 43 °C (group 1), or cycled for five times between room temperature and 43 °C (group 2), before being incubated at 37 °C for 4 h with the RED technique. Figure 5 shows the free fraction of three PBUTs for all groups. There was no observed difference in free fraction for any of the three PBUTs in both groups compared to controls, therefore temperature cycling does not appear to induce detectable persistent changes in albumin binding after 4 h.

#### 2.2.4. Hemocompatibility

Although no measurable effect on albumin binding was observed from repeated heating cycles, it is critical to ensure that this process does not cause adverse effects on whole blood, as different proteins and cell types may have varying thresholds for observable damage. For that purpose, to simulate the cyclical heating process of locally heating blood only in the dialyzer, blood samples were repeatedly heated for 30 s at different temperatures to simulate the blood transit time in the dialyzer. Samples were then analyzed for a range of hematology parameters (Supplementary Table S1) to assess cell damage, hemolysis, and inflammation markers.

**Figure 6:**
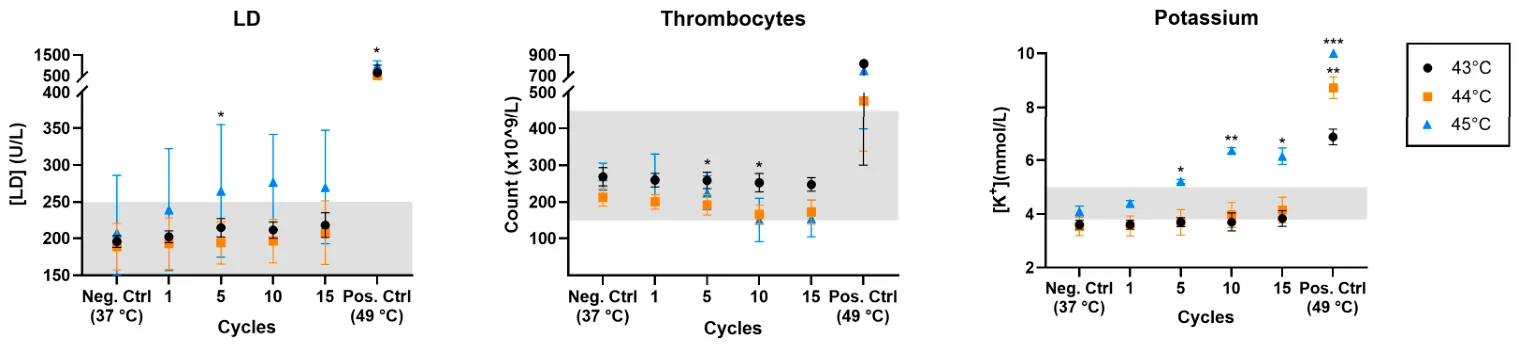
Blood integrity markers (lactate dehydrogenase (LD), thrombocyte count, and potassium) following repeated heating cycles at temperatures between 37 °C and 49 °C. The shaded gray region represents the physiological range. Data are presented as mean ± SD. Each temperature group (43, 44, and 45 °C) contains measurements from distinct groups of three donors each. Positive controls from the same donors as the 43, 44, and 45 °C samples are indicated by the corresponding symbols. Pairwise comparisons were made against the negative control of the same temperature group. * = *p* < 0.05; ** = *p* < 0.01; *** = *p* < 0.001.

Figure 6 shows three blood markers (thrombocyte count, lactate dehydrogenase (LD) and potassium) that indicate clotting and hemolysis, in response to different heating temperatures and number of heating cycles. Positive controls (49 °C) showed clear signs of cell damage in all three markers as all values sit outside the physiological range. Below 49 °C, all three markers demonstrate a cycle-dependent trend, with a decrease in thrombocytes with cycle count, and an increase in LD and potassium concentrations. After 15 cycles, thrombocyte counts decreased by 7.7 ± 2.1% at 43 °C and by 19.3 ± 6.2% at 44 °C compared to controls. Notably, at 45 °C, approximately half of the tested samples contained thrombocyte aggregates. In the positive control condition, the markedly elevated platelet counts are most likely attributable to small non-platelet particles or aggregates within the platelet size/scatter range being misclassified as platelets by the automated analyzer [36]. These values should be regarded as artifactual and not representative of the actual platelet concentration in the sample. LD concentrations increased by 11.8 ± 12.9%, 9.7 ± 8.2% and 33.9 ± 26.6% for 43 °C, 44 °C and 45 °C, respectively. Potassium levels increased by 6.4 ± 3.0%, 17.4 ± 4.0% and 50.2 ± 9.7% in the same temperature range. However, none of the above values for 43 °C and 44 °C were statistically significant given the sample size and high inter-donor variability. In addition, per-donor variability changed with both temperature and cycle count, indicating individual differences in response to thermal cycling (Supplementary Figures S1 and S2). Hemolysis rate after 15 cycles was 0.3% at 43 °C and 0.7% at 44 °C, falling under the 2% limit set by the ASTM F756-17 standard for hemolytic properties of materials [37], while increasing to 2.4% at 45 °C. No significant differences were observed in any of the other markers tested at 43 °C. Therefore, 43 °C appears to be a promising candidate for a safe heating temperature limit.

#### 2.2.5. Hemodialysis Mathematical Modeling

We explored the possible effect of increased PBUT free fraction in the dialyzer through increased temperature on dialysis adequacy parameters such as reduction ratio (RR) and monthly time-averaged concentration (TAC) using mathematical modeling. A two-compartment conventional HD model based on the work of Eloot et al. [38] was used, which includes the plasmatic and extraplasmatic compartments. Elevating temperature from 37 °C to 43 °C increased reduction ratios for IS, pCS, and HA by 15.4%, 7.9% and 3.7%, respectively; whereas, weekly TAC decreased by 12.3%, 5.7%, and 5.2% in the 5th week (Table 2).

**Figure 7:**
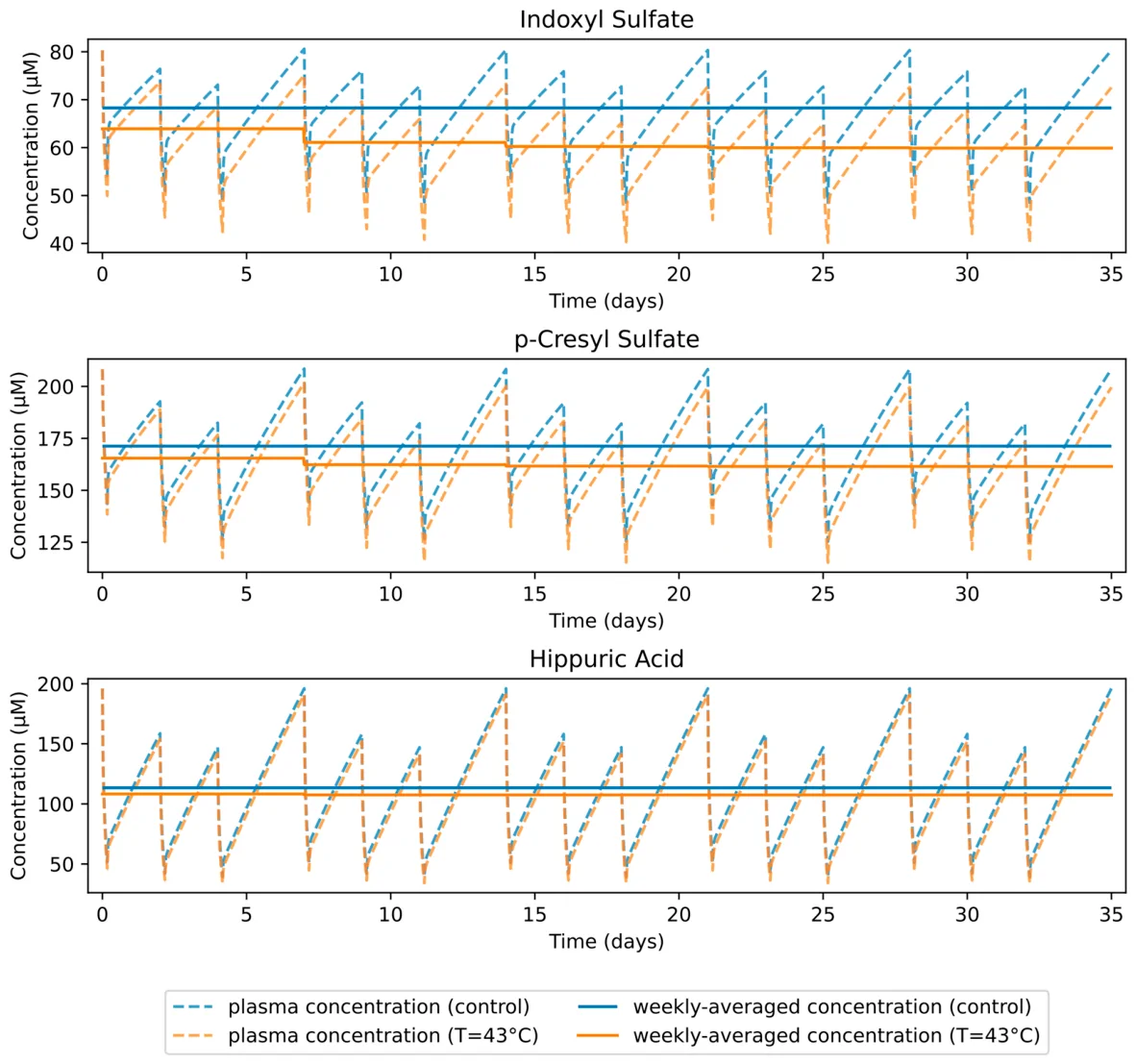
Calculated PBUT concentration in plasma over a 5-week period following initiation of heat treatment with a 3×/week 4 h hemodialysis schedule.

Figure 7 shows the simulated PBUT concentrations in plasma over a 5-week period with a 3×/week 4 h HD schedule, for control and increased temperature (T = 43 °C) conditions. Simulations start from the steady-state concentration of the control condition. Following the introduction of the elevated temperature condition, plasma concentrations gradually decrease, reaching a new steady state after ~3 weeks.

## 3. Discussion

We investigated two approaches, EM fields (A) and elevated temperature (B) to potentially increase the free fraction of three PBUTs. EM field application did not result in a measurable release of PBUTs, whereas elevated temperature significantly increased the free fraction of all three tested PBUTS.

**Figure 8:**
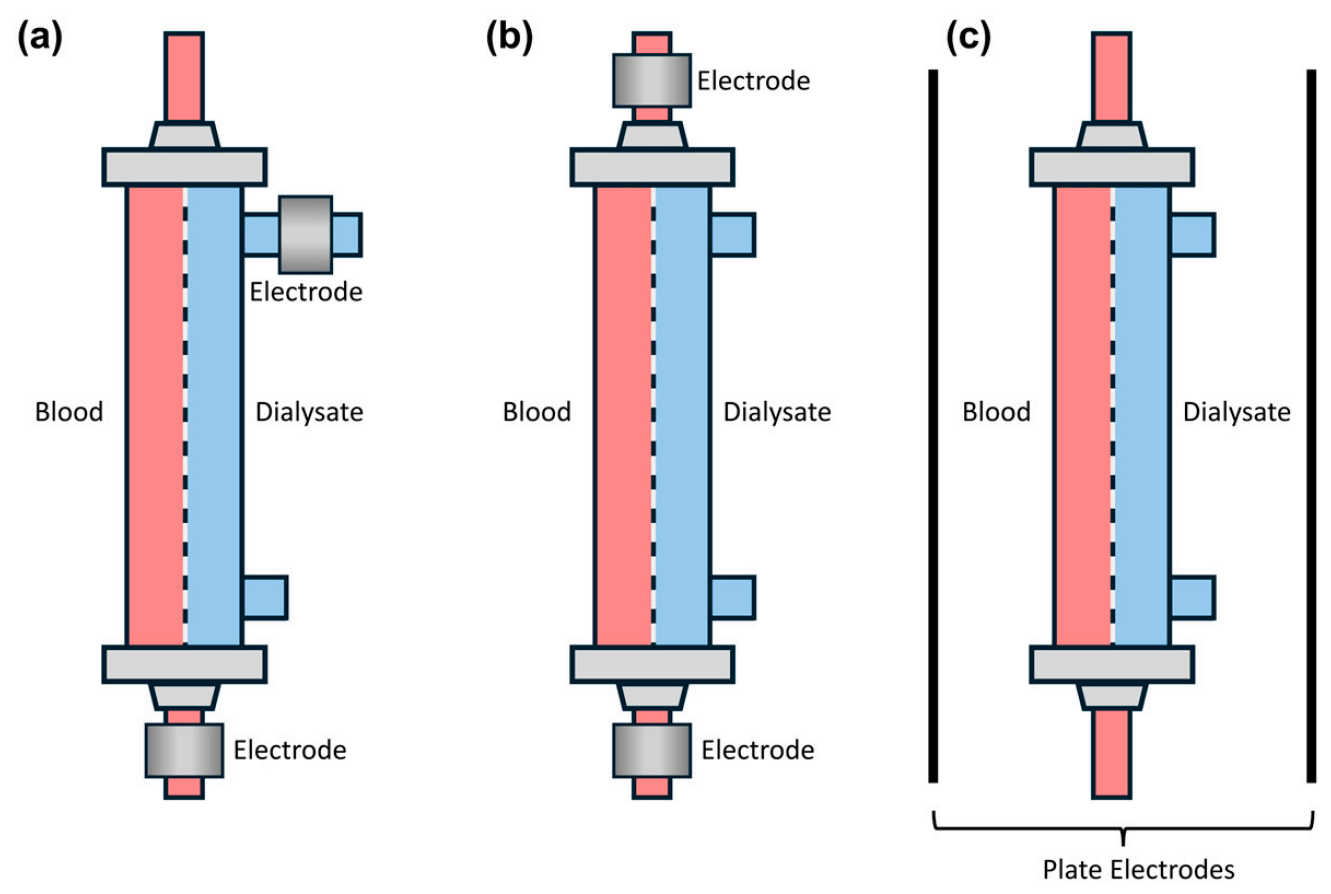
Electrode arrangements. (**a**) Blood–dialysate; (**b**) blood–blood; (**c**) parallel plates.

With EM fields, different electrode arrangements were tested, one of which was a parallel plate setup (see Section 5.1) comparable to a previously reported implementation [22]. To achieve the maximal PBUT release with EM fields, the electric field strength should ideally be concentrated near the dialyzer membrane pores. Hsiao et al. demonstrated a significant increase in p-cresol removal with localized, low-frequency electric fields (<1 Hz) emitted by the dialysis membrane, highlighting the importance of targeting albumin in close proximity to the membrane pores [23]. To also evaluate this approach, two additional setup arrangements were tested (Figure 8a,b), in which the electrodes were placed in direct contact with blood and/or dialysate to minimize boundary losses. However, in all three configurations, no effect on PBUT binding was observed. Similarly, Rodriguez-Chitiva et al. also found no significant effect on IS and pCS using a parallel-plate setup. However, no specific data on output power and frequency range were reported [39].

Several key differences between this work and the previously reported patent implementation [22] may explain the different outcomes in PBUT release using EM fields. Together with Prof. Jankowski, we investigated his original setup. The patented implementation of Jankowski et al. used single-pass dialysate flow and a static volume of albumin inside the dialyzer, which was then exposed to the EM fields, and PBUT concentration was only measured in the albumin volume (subtracting concentrations before and after exposure). Thus, PBUT removal was measured, while in this work only changes in free fraction were measured. The PBUT release that Jankowski et al. measured and attributed to the EM fields may have been enhanced by heat emitted by impedance-matching resistors in the tuning circuit. These resistors were positioned close to the dialyzer and became red-hot due to the amount of power dissipated in them. External temperature sensors on the dialyzer measured a temperature increase (unpublished data), but the albumin solution temperature *inside* the dialyzer was not measured and the EM field exposure time was not disclosed [22]. In the setup used in the current study, no temperature increase in fluids resulting from EM fields (P = 4 W) was observed at any of the applied frequencies (1–600 MHz).

In contrast to the application of EM fields, elevated temperature resulted in PBUT displacement within the range predicted by theoretical modeling of free fraction, even surpassing the predicted response for HA (Figure 4). This disparity may be caused by the model assumptions not holding under experimental conditions, such as varying enthalpy values in this temperature range [31,32,33,34], PBUT binding to two binding sites with different binding affinities (Table 1), and binding competition between different PBUTs within the samples. In addition, samples were spiked with PBUT concentrations, which can lead to binding site saturation, and the binding characteristics of HSA may alter at 35 °C and 42 °C due to reversible conformation changes, as previously reported [40,41,42]. The two-compartment HD model was used to evaluate the possible impact of an increased free fraction on PBUT plasma concentrations over time, predicting a decrease in the average concentration for all three tested PBUTs after a month of treatment. Importantly, these model-based predictions do not replace direct PBUT measurements in patients, and are therefore not indicative of clinical efficacy.

Temperature-based PBUT removal enhancement was demonstrated to be less effective than chemical displacers, which have been reported to increase the free fractions of IS and pCS by up to 4-fold [1,12,14,15]. However, the non-invasive, temperature-based method has several advantages compared to other PBUT removal methods such as chemical displacers, ionic strength increases and adsorbents. The approach is relatively straightforward as the current HD setup can be used without requiring infusion or addition of components into the blood or dialysate circuit. A key requirement for the application of local heating in the dialyzer as an add-on module is rapid PBUT binding kinetics, as the PBUT free fraction must be affected in a time interval similar to that of the blood’s transit time in the dialyzer (~20 s). The in vitro dynamic setup in the present study (Figure 3a) demonstrated that IS concentration in dialysate responds to temperature changes in the dialyzer in <8 s, when accounting for the measurement delay of ~3 s from the dialysate reaching the in-line IS sensor. On a molecular level, parameters such as the mean lifetime of the PBUT binding complex can be estimated from binding reaction kinetics [43,44]. The maximum estimated free fraction re-equilibration times are short (<1 s) for IS and pCS, obtained through previously published fitted HD model data and binding constants [34,45,46,47]. In addition to increasing the PBUT free fraction, elevated temperature treatment may also improve dialytic toxin removal by reducing blood viscosity, thereby enhancing diffusion across the dialysis membrane [48]. Furthermore, heating can be quickly discontinued without further intervention since the protein binding effect is reversible as demonstrated in this study. The PBUT releasing effect is therefore confined to the extracorporeal blood circuit and does not cause a systemic increase in the free fraction, unlike chemical displacers, whose effects persist in the body until they are metabolized. This systemic increase in the free PBUT fraction with displacers may lead to adverse effects [49]. Therefore, despite its more modest effect on PBUT displacement, the relative simplicity and locally confined, rapidly reversible nature of extracorporeal heating may warrant further investigation, although its long-term safety and clinical efficacy remain to be established.

Notably, heating may also reduce the protein binding of drugs and their metabolites, potentially increasing their free fraction, dialytic clearance, and altering their pharmacokinetic profiles in patients undergoing HD. In this population, uremia-related changes in protein binding could be compounded by heat-induced dissociation of drug–albumin complexes. For highly protein-bound drugs, whose dialytic removal is normally limited, modest increases in the free fraction may increase dialytic removal and thereby reduce systemic drug exposure, creating a risk of subtherapeutic concentrations. This is an important distinction from chemical displacers, in which an increased systemic free fraction may instead raise concern for enhanced pharmacologic effects and toxicity [50]. Clinically relevant examples of drugs encountered in the dialysis population include warfarin, phenytoin, valproate, furosemide, antimicrobials such as ceftriaxone, and direct oral anticoagulants. Although dialysis-related drug dosing is scheduled around treatment sessions, thus making the interaction relatively predictable, dedicated pharmacokinetic studies may be required before its clinical implementation to determine whether dose or timing adjustments are necessary. Moreover, the same mechanism may affect endogenous albumin-bound ligands, including bilirubin, free fatty acids, tryptophan, thyroid hormones, and protein-bound calcium, with potential physiological consequences that also warrant investigation. Restricting the heating effect to the dialysis filter, thereby promoting dissociation primarily within the extracorporeal circuit, would minimize systemic changes in protein binding and therefore may be more advantageous than adding displacer molecules that also elevate the intracorporeal free fraction.

In addition, the heating module is also compatible with, and additive to all HD modalities covered by the international standard IEC 60601-2-16, such as HDF and HDx. In pre-dilution HDF, the substitution fluid will impact the thermal balance and needs to be accounted for to achieve the desired blood temperature inside the dialyzer. In post-dilution HDF, the substitution fluid may help in further cooling the blood to ~37 °C before returning to the patient, lowering the requirements of a dedicated cooler.

An important question is whether the increase in temperature is hemocompatible and what the highest temperature is that can be applied. Most available data on blood heating address blood transfusions, where blood is heated from 2 to 6 °C storage temperature to 37 °C before infusion [51]. A recent systematic review with meta-analysis of 17 studies reported that blood heating is safe up to 46 °C, as it does not cause clinically significant hemolysis [52]. Furthermore, the standard IEC 60601-2-16, describing requirements for HD equipment, allows a dialysate temperature up to 46 °C for short periods as blood temperatures up to 46 °C in the extracorporeal circuit have been used for hyperthermia treatment [53]. The initial exploratory findings from the current study indicated no pronounced hemocompatibility issues at temperatures up to 43 °C under the conditions tested, but the significant adverse effects found at 45 °C highlight the need for further testing and for a precise temperature control given the change in outcomes from a small temperature range.

The experiments had some notable limitations that should be acknowledged. With the EM field setup, only MHz-range fields were tested, as these represent most known effects on albumin binding in the literature. Recently, GHz-range fields have also been demonstrated to affect binding [54], but this frequency range was out of scope for this work since it was not technically feasible. Therefore, this approach remains a promising avenue for future studies and additional optimizations. The temperature experiments to characterize PBUT binding had the limitation of subjecting samples to >30 min of elevated temperature (>37 °C) in both static and dynamic in vitro setups, whereas in HD the blood would undergo much shorter, but repetitive heating cycles when passing through the dialyzer. The test samples used either infusible albumin, which contains high concentrations of stabilizers that impact binding, or purified albumin, which contains primarily only the spiked PBUTs as binding compounds. These albumin types may present different binding responses to either EM fields or temperature than albumin in uremic plasma, which suffers from uremia-induced post-translational modifications in chronic kidney disease (CKD) [34,55]. Further studies should therefore be performed using uremic plasma to reproduce the complex binding environment found in physiological conditions. In addition, albumin functional integrity was assessed indirectly through the binding experiments, and not directly using an analytical technique such as HPLC or a dedicated structural characterization method. However, the comparable binding behavior observed across all tested conditions indicates that albumin retained its ligand-binding capacity, suggesting that no substantial conformational changes affecting the binding site occurred under the experimental conditions. Notably, the higher effect of heating seen in Figure 3b in the experiments containing stabilizers, which was also observed by Raillon et al. [15], suggests a synergistic effect that warrants further investigation. The hemocompatibility tests required heating the sample tubes for 1.5 min until they reached the desired temperature, increasing the exposure times to higher temperatures than the required 20 s. In an HD setup, faster heating could be achieved with commercially available blood heaters, for instance. In addition, the negative control samples were exposed to the 37 °C water bath for the duration of the experiments. As a result, the true baseline levels in unheated blood were not measured, so some of the observed changes may reflect a baseline change in analyte levels due to handling or prolonged exposure at 37 °C, rather than purely the effects of elevated thermal treatment. Furthermore, the ASTM F756-17 standard specifies that the hemolytic index must be calculated directly by measuring free plasma hemoglobin, whereas it was calculated indirectly by the change in potassium concentration in this work. Donor groups were different for each temperature condition, so inter-donor variability may have confounded the evaluation of temperature effects. Finally, blood marker measurements were performed on the same day as the experiment, so possible latent effects such as changes in thrombocyte morphology are unknown. However, even with additional delayed measurements, the cumulative significance of the observed changes remains uncertain. Although small per-session effects may be acceptable, repeated exposure to low-grade hemolysis and platelet reductions over months or years could contribute to anemia burden, altered erythropoiesis-stimulating agent (ESA) requirements, and other downstream consequences in patients who already suffer from uremic platelet dysfunction and are frequently anticoagulated. Conventional HD has been shown to cause up to 6.2% reduction in thrombocyte counts post-session [56], which is comparable to the change observed in this study. However, longer-term studies are needed to determine whether these small per-session effects accumulate with repeated treatment and if they are additive to the effects already seen with conventional HD.

An important consideration when interpreting PBUT removal methods is that circulating toxin concentration remains a surrogate endpoint. Although several approaches can reduce levels of protein-bound uremic toxins, it has not yet been established that such reductions translate into improved patient outcomes. Indeed, clinical trials of oral adsorbents, which demonstrated a significant reduction in IS levels, have been largely neutral [57]. Furthermore, the predicted reduction in PBUT concentrations achieved by local heating is modest and may be comparable to normal intra-individual variability related to diet, microbiome and residual kidney function. Consequently, the clinical relevance of a reduction of this magnitude remains uncertain and may be limited.

This uncertainty does not, however, negate the rationale for further investigating temperature as a strategy to enhance PBUT removal. Given the biological plausibility and observational associations linking PBUTs with adverse outcomes, together with the fact that these solutes are efficiently cleared by the native kidneys, improving their removal during dialysis may remain a relevant therapeutic target. Importantly, local heating may offer practical advantages over other strategies aimed at increasing the free fraction of PBUTs. Many existing approaches are invasive and increase the free fraction systemically, whereas this technique offers the possibility of transiently increasing the free fraction specifically within the dialyzer, thereby avoiding increased systemic exposure to unbound toxins. Moreover, implementation could potentially remain close to conventional hemodialysis practice with a reusable add-on with limited recurring costs and only modest additional treatment complexity, although appropriate temperature monitoring and safety measures would be required. Previous work demonstrating the feasibility and safety of controlled extracorporeal blood heating further supports exploration of this approach [53].

## 4. Conclusions

In conclusion, this study evaluated the efficacy of two non-invasive methods for enhancing PBUT removal during HD. While EM fields showed no measurable effect, elevated temperatures emerged as a simple and cost-effective approach capable of reversibly increasing the free fraction of PBUTs. This method holds realistic potential for clinical translation, as it is in principle combinable with all existing HD devices. The proposed method requires further validation, particularly with regard to long-term hemocompatibility and safety.

## 5. Materials and Methods

All reagents were purchased from Sigma Aldrich (St. Louis, MO, USA) unless otherwise mentioned. HSA solution for infusion (Albuman 40 g/L) was purchased from Prothya Biosolutions BV (Amsterdam, The Netherlands). Dialyzers (Polyflux 2H, 0.2 m^2^) were purchased from Baxter (Glenview, IL, USA). Dialysate was freshly prepared using Milli-Q water, sodium bicarbonate and Dirinco 831 acid concentrate (Oss, The Netherlands). Ammonium formate was purchased from HoneyWell (Morristown, NJ, USA) and HPLC-grade methanol was purchased from Biosolve (Valkenswaard, The Netherlands). Fresh whole blood was obtained from healthy human donors.

### 5.1. Approach A: EM Fields

An infusible HSA solution (40 g/L, 250 mL) spiked with IS (for a final concentration of 60 µM) was dialyzed against an equal volume of dialysate and both sides were recirculated. The non-bound IS concentration in the dialysate therefore reaches an equilibrium, after which the test conditions were performed. The used IS concentration is lower than the average uremic concentration (~150 µM) in order to stay within the detection range of the real-time in-line IS sensor. In addition, samples were taken on both reservoirs before starting each experiment, after equilibrium of IS levels in dialysate, and at the end of the experiment. These samples were stored at −20 °C until analysis. The free fraction was calculated as follows:

(1) $$\%\;\mathrm{PBUT}\;\mathrm{Free}\;\mathrm{Fraction}=\frac{C_{\mathrm{dialysate}}}{C_{\mathrm{HSA}}}\cdot 100$$

where *C_HSA_* = *C_spiked_* − *C_dialysate_* when the system is in equilibrium.

Three different electrode arrangements were tested. In the first two, stainless steel rings (width = 4 cm) were placed in-line with the tubing and in direct contact with the circulating fluids (Figure 8a,b). These were placed next to the dialyzer inlets and outlets, in both a blood–dialysate and blood–blood configuration. The third arrangement consisted of rectangular copper-plate electrodes arranged in parallel (7 × 7.5 cm), 3 cm apart (equal to dialyzer diameter; Figure 8c).

The EM signal was applied at distinct frequencies and in sweeps (signal generator: Rohde & Schwarz SMB 100A (Munich, DE, Germany); power amplifiers: Kalmus 210LC (Bothell, WA, USA) and TESEQ CBA 9477B (Luterbach, CH, Switzerland)), with an input power up to 26 dBm (4 W). Individual frequencies of 1, 2, 3, 100, 110, 111, 112, 113, 114, 115, 135, 140, 141, 142, 143, 185, 225, and 300 MHz were applied for a minimum of 5 min each, based on previous reports demonstrating their effectiveness [19,21,22]. Additional frequency ranges were tested using frequency sweep settings with dwell times of >5 s at each frequency, as described in Table 3. The exposure time to EM fields of albumin crossing the dialyzer was ~5 s, given the dialyzer’s internal blood volume (17 mL) and the used albumin flow (200 mL/min).

**Figure 9:**
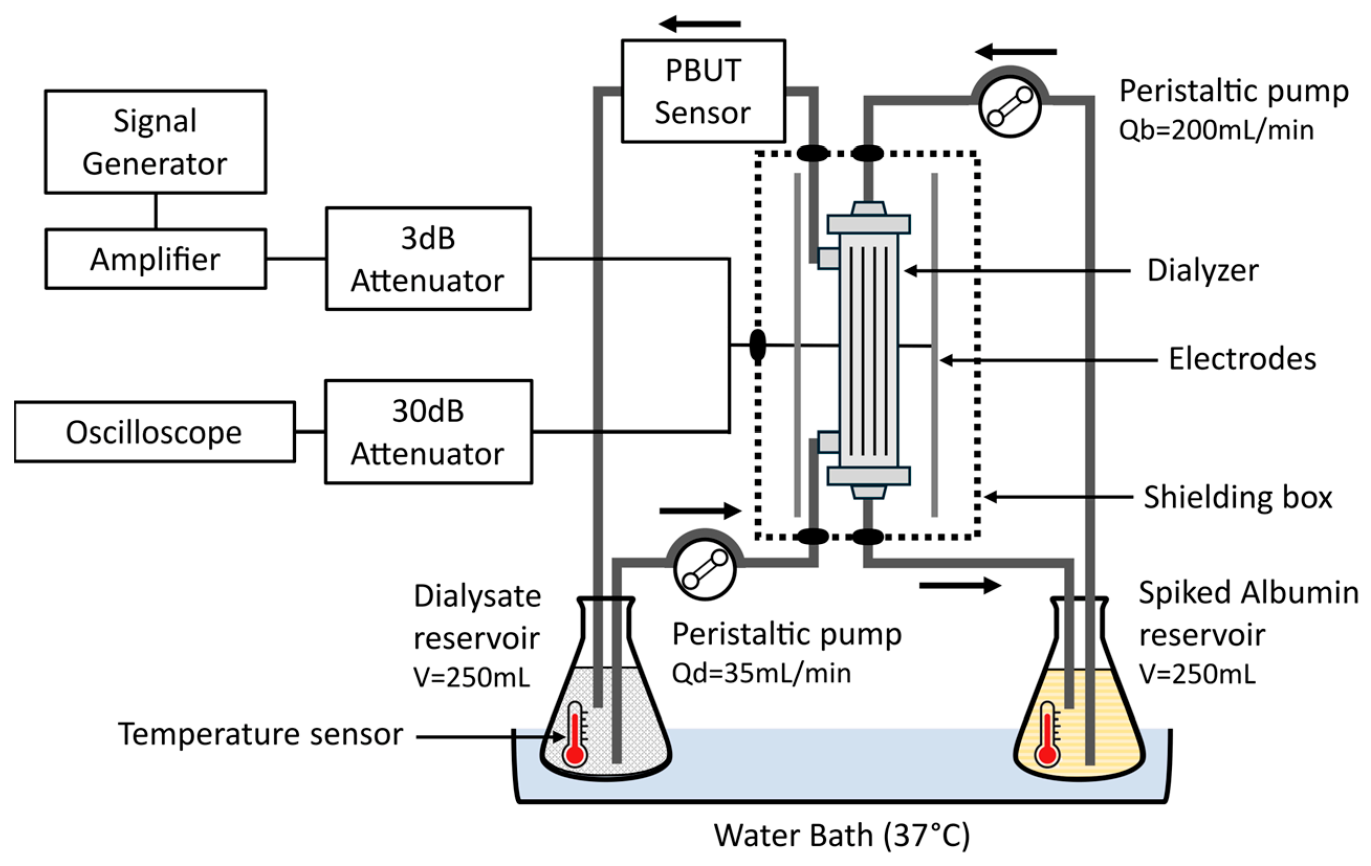
EM field experimental setup diagram. Qb—albumin flow; Qd—dialysate flow.

**Figure 10:**
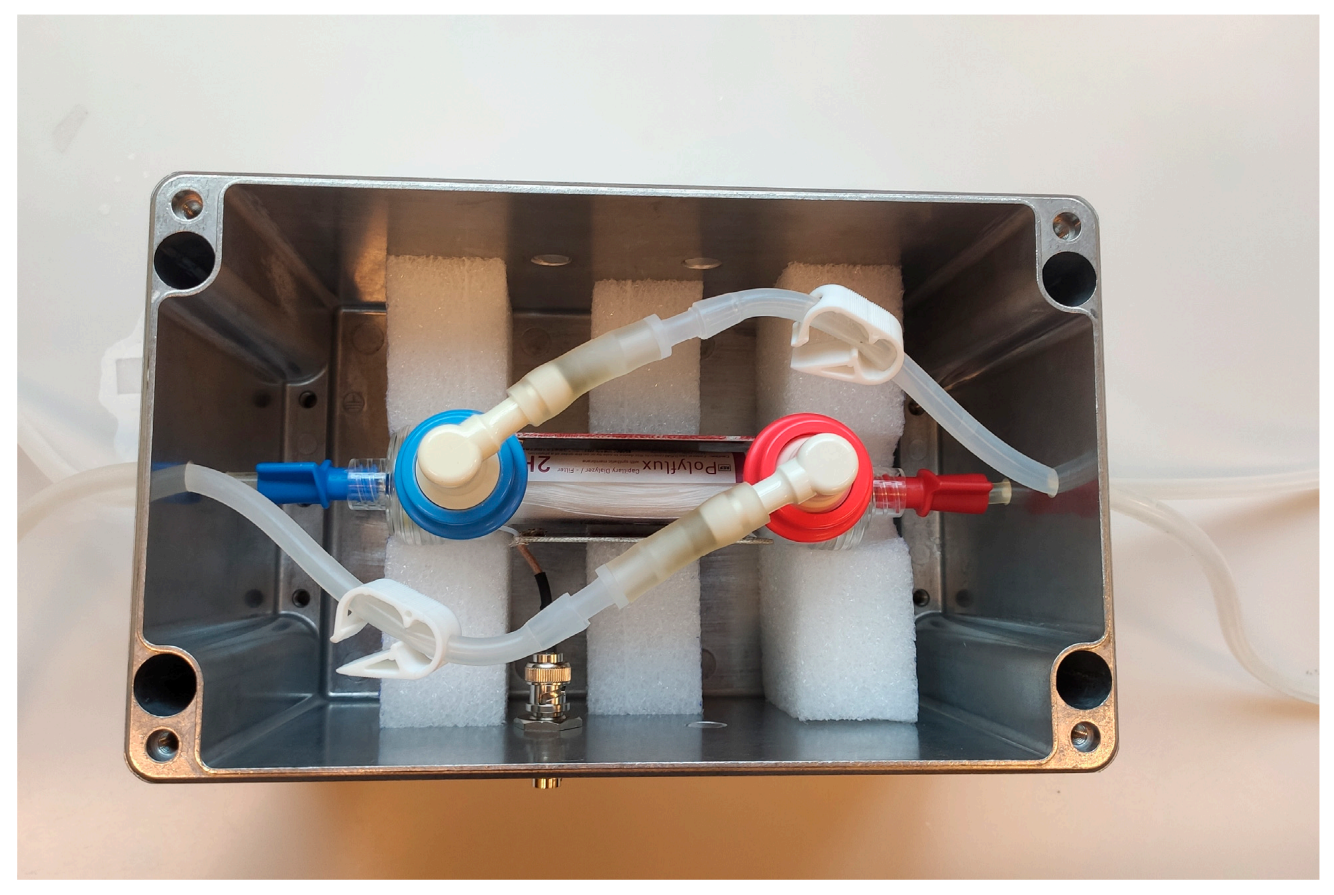
Parallel plate and dialysis setup in (here opened) shielding enclosure.

Experiments were performed at room temperature and 37 °C to better simulate physiological conditions. The fluids were maintained at 37 °C by placing both albumin and dialysate reservoirs in the same water bath (Thermo Scientific Precision, Waltham, MA, USA) and temperature was monitored at the dialyzer inlet of each circuit (Sefram 9816B, Saint-Étienne, France) (Figure 9). The circuit’s silicone tubing (ID = 3.2 mm, OD = 4.8 mm, RS Components B.V., Haarlem, The Netherlands) was insulated to minimize heat losses before reaching the dialyzer, and the dialyzer–electrode arrangement was placed inside an aluminum enclosure to reduce EM signal emissions towards the environment and prevent interference with nearby equipment (Figure 10).

### 5.2. Approach B: Temperature

#### 5.2.1. Model

The change in PBUT free fraction from a temperature change was estimated with Equation (2), given a known free fraction at the starting temperature and the enthalpy change of the reaction [24].

(2) $$f_{u}^{o}\left(T_{2}\right)=\frac{1}{1+\left(\frac{1}{f_{u}\left(T_{1}\right)}-1\right)\exp\left(\frac{{∆H}^{o}\left(T_{2}-T_{1}\right)}{RT_{1}T_{2}}\right)}$$

where

- $f_{u}^{o}\left(T_{2}\right)$—PBUT free fraction at the desired temperature;
- *f_u_*(*T*_1_)—PBUT free fraction at the starting temperature;
- *T*_1_—Starting temperature (K);
- *T*_2_—Final temperature (K);
- ∆*H^o^*—Enthalpy change of the reaction for each binding site (∆*H^o^* > 0 for dissociation);
- *R*—Gas constant (8.3145 J⋅K^−1^⋅mol^−1^).

This model has the following assumptions:

- There is an excess of HSA versus the bound PBUT.
- All n binding sites of HSA have the same affinity.
- ∆*H^o^* is constant in the desired narrow temperature interval.
- Constant ambient pressure.

#### 5.2.2. In Vitro Dialysis Setup

**Figure 11:**
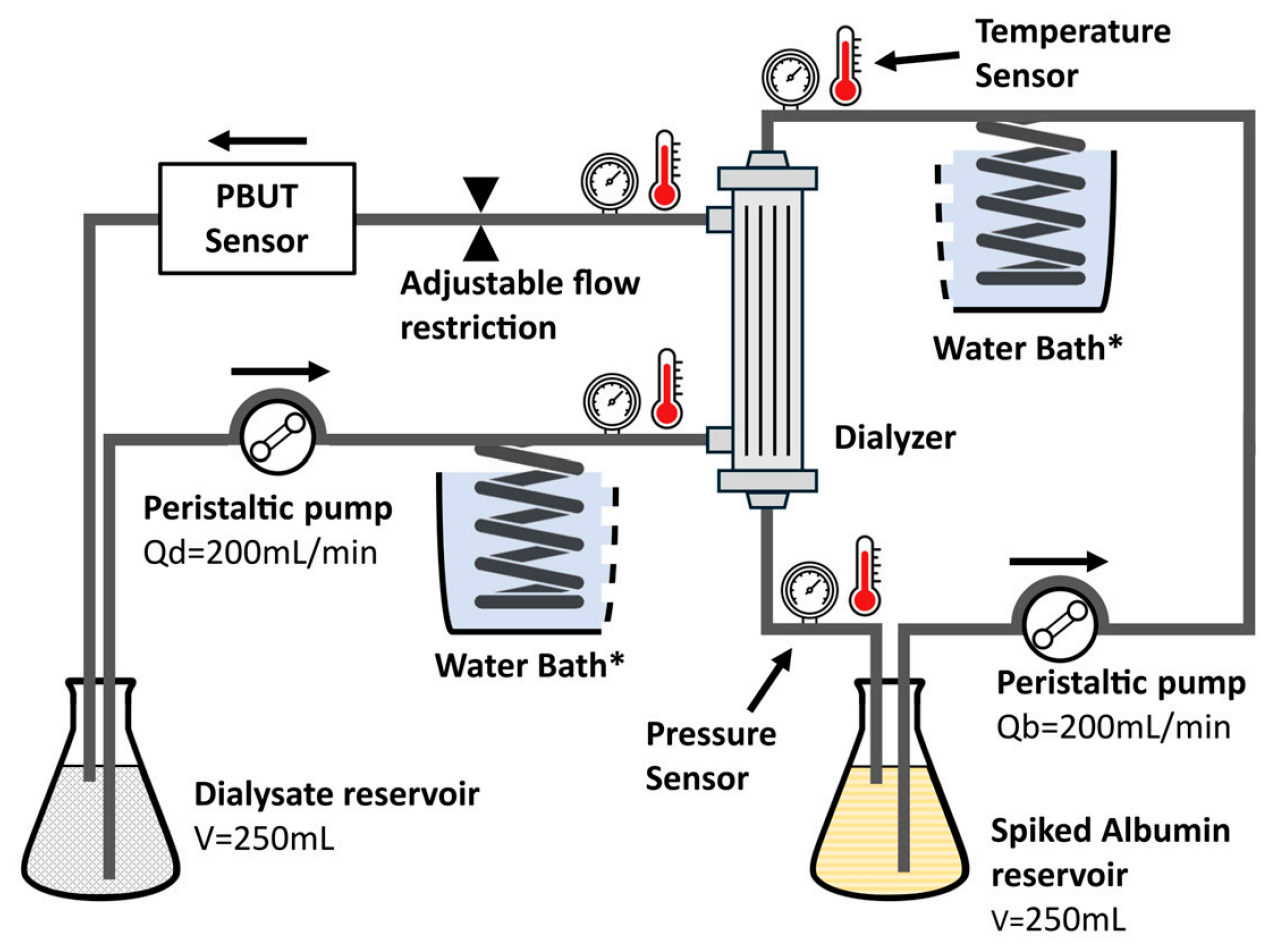
In vitro dialysis setup for elevated temperature tests. Qb—albumin flow; Qd—dialysate flow. * Both coils were placed in the same water bath.

To explore the effect of temperature, the experimental setup and protocol were similar to those mentioned above for EM fields, but without RF-related components (electrodes, amplifiers and shielding; see Figure 11). Transmembrane pressure (TMP) was controlled with a flow and pressure control platform (Inspector Membrane Distillation Pilot, DEMCON Convergence BV, Enschede, The Netherlands). This platform uses 4 pressure sensors (Type PR-33X, Keller Pressure, Winterthur, Switzerland) on the albumin and dialysate side to calculate the TMP and a flow restrictor to prevent ultrafiltration over time.

The circulating fluids were heated through a coiled section of silicone tubing (ID = 3.2 mm, OD = 4.8 mm, RS Components B.V.) submerged in a water bath which ensured uniform heating and minimized hotspots. Both the albumin and dialysate circuit were heated in the same water bath so that the dialyzer inlet temperature was identical for both sides of the filter. Temperature was measured in all four dialyzer connections (both inlets and both outlets).

The experiment was started at room temperature until IS levels in the dialysate reached equilibrium, then fluid temperature was increased to 30 °C and maintained until a new equilibrium in dialysate was reached. The process was repeated at 35 °C and 40 °C, and then in reverse order at the same points back down to the initial temperature. Samples were also taken from both reservoirs (1) before starting each experiment, (2) after equilibrium of IS levels in dialysate at each temperature step, and (3) at the end of the experiment. These samples were stored at −20 °C for analysis. Experiments were performed with *n* = 2 for infusible albumin and *n* = 1 for purified albumin.

#### 5.2.3. Rapid Equilibrium Dialysis (RED)

**Figure 12:**
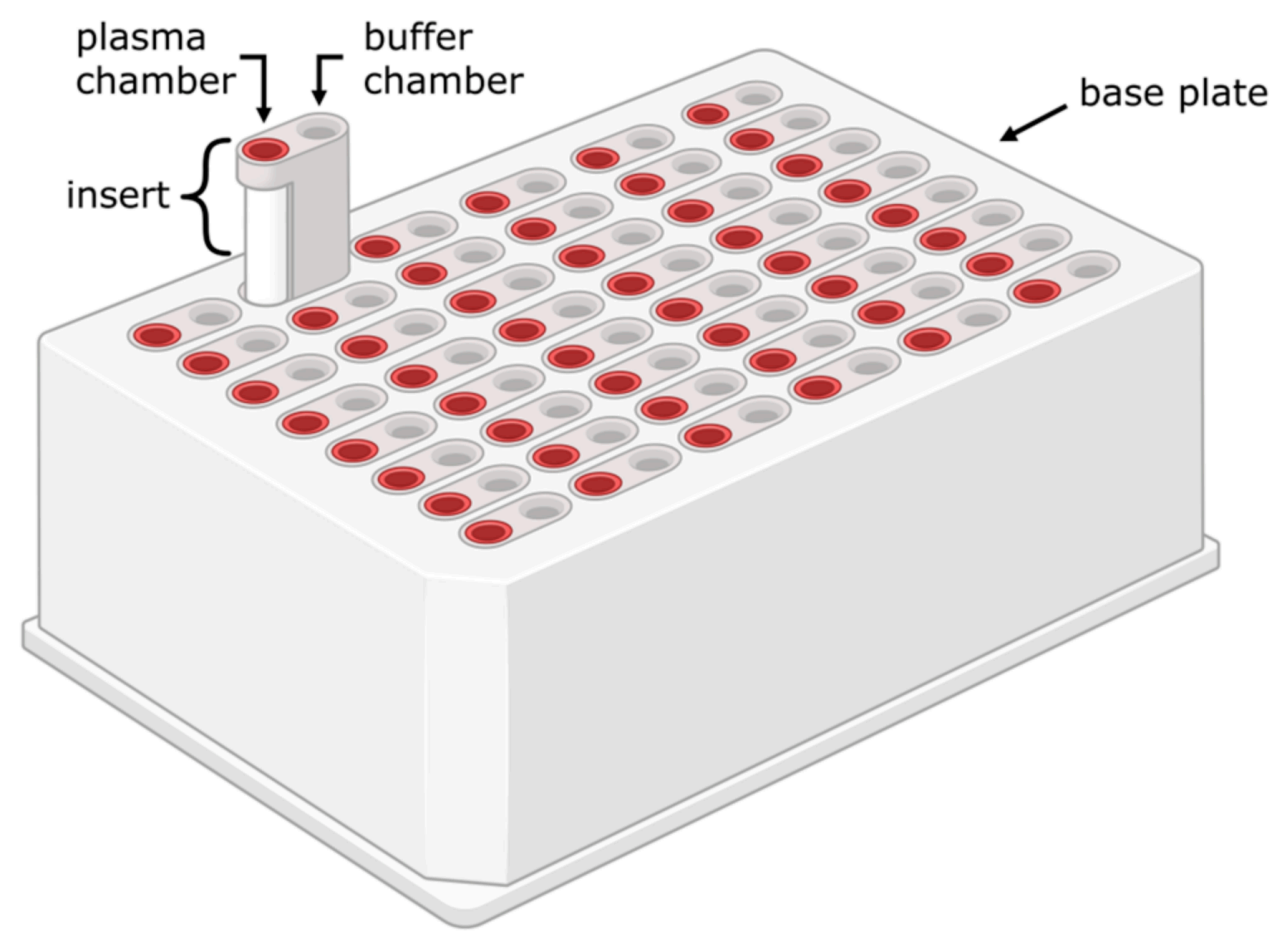
The rapid equilibrium dialysis (RED) device. Reproduced without modification from [7] (under a CC BY 4.0 license).

Static dialysis experiments were performed using single-use rapid equilibrium dialysis (RED; Thermo Fisher, Rockford, IL, USA) cartridge inserts (Figure 12). A RED insert is composed of two chambers (sample and buffer), separated by a 12 kDa MWCO cellulose dialysis membrane. A purified HSA solution (40 g/L; from lyophilized powder), reported to contain a total of ~540 µM fatty acids [58] was dissolved in PBS and used for the sample side (V = 500 µL). Both solutions were spiked with PBUTs to achieve final concentrations of 150 µM IS, 200 µM pCS and 400 µM HA to simulate uremic conditions [8]. PBS was used for the buffer side (V = 750 µL). The samples were incubated for 4 h with an orbital shaker at 200 rpm, in an oven at temperatures between 35 and 45 °C, and finally stored at −20 °C until HPLC analysis. All test conditions were performed in quadruplicate. PBS samples spiked with PBUTs were used as controls to ensure equilibrium was reached after 4 h.

#### 5.2.4. Hemocompatibility

Assuming 5 L of blood volume for the average dialysis patient and an HD blood flow of 300 mL/min, the total blood volume passes though the dialyzer ~15 times in a 4 h HD session, or once every ~15 min. In addition, the blood’s transit time though the filter is approximately 20 s, assuming a dialyzer internal blood volume of 100 mL. To simulate the cyclical heating process of locally heating blood only in the dialyzer, blood samples were maintained at 37 °C and then repeatedly heated for 30 s to simulate the blood transit time in the heated dialyzer, at temperatures up to 49 °C, after which they were cooled back to 37 °C. Heating and cooling was performed in separate shaking water baths. Due to experimental time constraints, the interval between each heating cycle was reduced from 15 min to 5 min. Each sample was split into 200 μL PCR thin-walled tubes to ensure rapid heating and cooling, after which they were pooled again for analysis. Samples maintained at 37 °C during the entire experiment acted as negative controls and samples heated once to 49 °C acted as positive controls for blood damage indicators. Immediately after testing, samples were analyzed for a range of hematology parameters (see Supplementary Data) at the Central Diagnostics Lab (CDL) of UMC Utrecht to assess cell damage, hemolysis, and inflammation markers. The percentage change in each marker from the heating process was calculated for each donor individually. The mean percentage change and standard deviation were then calculated across donors.

Hemolysis rate was calculated indirectly from potassium concentrations in plasma (Equation (3)) [59,60].

(3) $$\%\;\mathrm{Hemolysis}\;\mathrm{rate}=\frac{\left({\left[K^{+}\right]}_{\mathrm{cycled}}-{\left[K^{+}\right]}_{\mathrm{control}})\cdot (1-\mathrm{Hct}\right)}{{\left[K^{+}\right]}_{\mathrm{RBC}}\cdot \mathrm{Hct}}\cdot 100$$

where ${\left[K^{+}\right]}_{\mathrm{RBC}}$ = 100 mM and hematocrit (Hct) = 0.46 (sample average).

#### 5.2.5. PBUT Binding Reversibility

For the PBUT binding reversibility tests, temperature cycling was performed using a setup similar to that used for the hemocompatibility tests. Here, PBUT-spiked albumin samples were used instead of blood (IS, pCS and HA at uremic concentrations). Samples were either kept at 37 °C (control), cycled for five times between 37 °C and 43 °C (group 1), or cycled for five times between room temperature and 43 °C (group 2), before being incubated at 37 °C for 4 h with the RED technique. The samples were finally stored at −20 °C until HPLC analysis.

#### 5.2.6. Hemodialysis Mathematical Modeling

**Figure 13:**
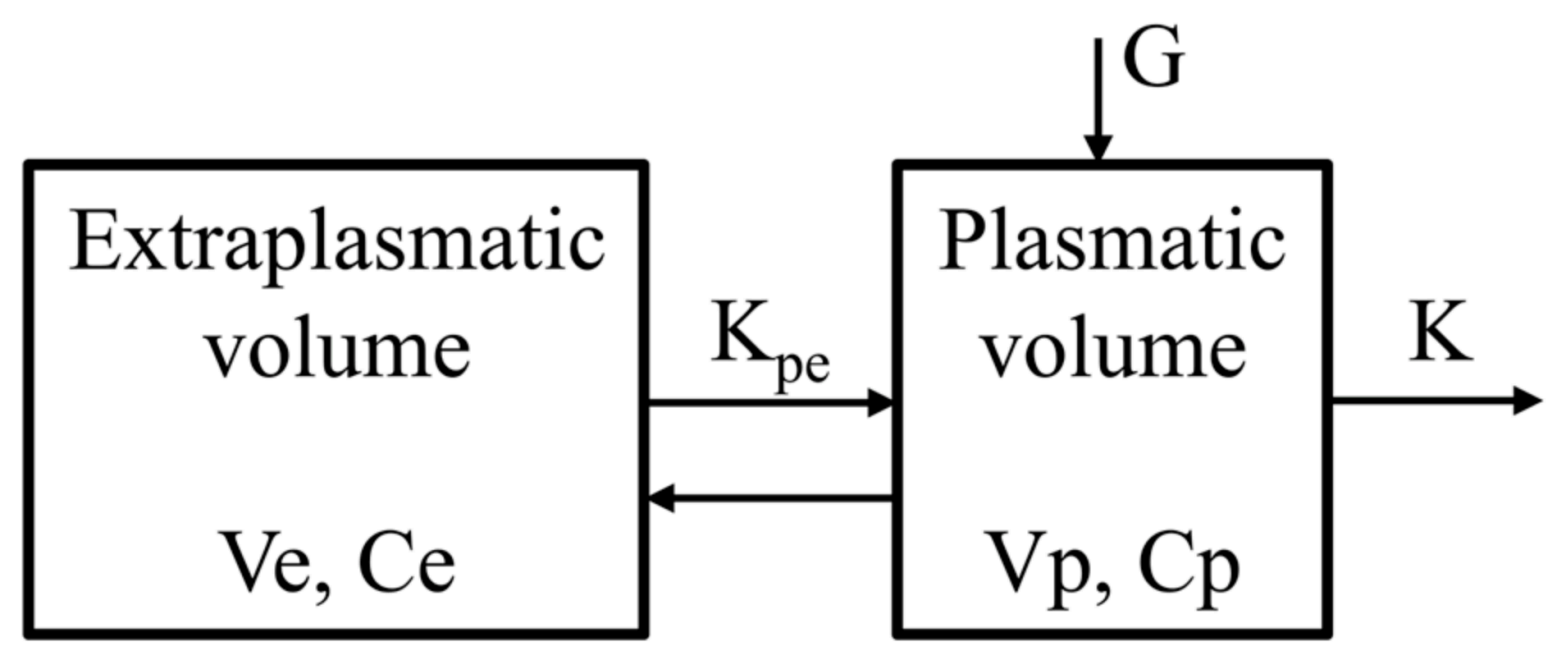
Two-compartment hemodialysis model. V: compartment volume; C: toxin concentration; K: dialyzer clearance; Kpe: intercompartment clearance; G: toxin generation. Adapted from [38] (under a CC BY 4.0 license).

A two-compartment conventional HD model was implemented in Python (version 3.13.11) based on the work of Eloot et al. [38], which includes the plasmatic and extraplasmatic compartments (Figure 13).

The method of heating was assumed to alter protein binding only within the extracorporeal circuit, so patient-specific kinetic parameters, including compartment volumes, intercompartmental clearance, and toxin generation, were retained from the validated base model (Table 4). Ultrafiltration rate (0.41 L/h) was also taken from the same work.

The effect of increased free fraction from heating was represented exclusively by modifying the dialyzer clearance. Since toxin clearance inversely correlates to protein binding [38], the clearance of each toxin at elevated temperature was calculated using the linear relationship between free fraction and clearance reported by Eloot et al. (R^2^ = 0.956) [38]. The original clearance of a toxin in the HD model was used as the reference clearance, corresponding to its baseline free fraction. The slope of the linear regression was then used to estimate the change in clearance associated with the increase in free fraction induced by heating according to the in vitro experiments in this work (Figure 4). To preserve the original model’s clearance at baseline, the regression relationship was anchored at the baseline free fraction and clearance. This approximation was considered valid as this model uses a lumped PBUT clearance, which does not differentiate between free and bound fractions, but only total removal.

Dialysis adequacy parameters such as reduction ratio and weekly time-averaged concentration (TAC) were calculated for IS, pCS, and HA. Compartment volume refill rate between HD sessions was set to linear and adjusted in the inter-session period so that predialysis volumes were constant. A 5-week window was used to calculate weekly TAC values. Toxin concentrations over time were determined by solving the mass balance equations for both compartments (Equations (4) and (5)):

{(4)Vp⋅dCpdt+Cp⋅dVpdt=G−K⋅Cp+Kpe⋅Ce−Cp(5)Ve⋅dCedt+Ce⋅dVedt=−Kpe⋅Ce−Cp

### 5.3. In-Line Analysis of IS

For IS detection, a real-time in-line optical sensor (Optofluid Technologies, Tallinn, Estonia) was used (detection range: 0.5–15 µM). This UV-based sensing method was previously described by Fridolin et al. [61,62]. The in-line sensor’s response is temperature dependent, where a higher temperature results in a lower measured signal. A correction curve was obtained and verified from tests performed in triplicate in the temperature range of 18–46 °C. This correction factor was then applied as post-processing to the measured signal according to the dialysate outlet temperature.

### 5.4. HPLC Analysis of PBUTs

#### 5.4.1. Sample Preparation

A 1:4 ratio of cold (4 °C) acetonitrile (ACN) was added to all albumin-containing samples to precipitate the protein. Samples were then vortexed and centrifuged (10,000 rcf for 10 min at 4 °C). The supernatant was transferred to a new tube and evaporated with a vacuum concentrator. The resulting pellet was reconstituted in purified water and finally filtered with a 0.22 µm filter. The non-albumin-containing samples from the buffer side were spun down (10,000 rcf for 10 min at RT) before HPLC injection.

#### 5.4.2. Analysis

PBUT concentrations were determined with reverse-phase HPLC (Arc HPLC System; Waters (Milford, MA, USA)) equipped with a C8 column (XBridge^®^ Ethylene Bridged Hybrid 2.5 µm 4.6 × 150 mm; Waters), a fluorescence detector (2475 FLR) and a UV detector (2998 PDA). IS and pCS were measured on FLR channel, with excitation and emission wavelengths of 272/374 nm for IS and 264/290 nm for pCS, and HA was measured on the UV channel (λ = 245 nm, spectral bandwidth = 4.8 nm). The eluents were composed of a 50 mM ammonium formate buffer adjusted to pH 3.0 with HCl, and methanol. Eluent A consisted of ammonium formate and methanol with a ratio of 90:10 (*v*/*v*), while eluent B had a ratio of 10:90 (*v*/*v*). The gradient elution was programmed as follows: eluent B was increased linearly from 30 to 50% for 3 min after injection. This step was followed by flushing the column with 100% solvent B for 2 min, and, finally, the column was reconditioned back to the starting condition of 30% solvent B, resulting in a total run time of 8 min per sample. The samples were eluted at 40 °C with a flowrate of 1 mL/min and injection volume of 50 µL.

The free fraction from the RED experiments was calculated as follows (Equation (6)):

(6) $$\%\;\mathrm{PBUT}\;\mathrm{Free}\;\mathrm{fraction}=\frac{c(\mathrm{buffer}\;\mathrm{chamber})}{c(\mathrm{sample}\;\mathrm{chamber})}\cdot 100$$

Calibration curves were freshly prepared and quality control (QC) samples for all PBUTs were included. Inter-day variability of QC samples was <2.5%. PBUT concentrations were calculated using Empower^®^3.10 software.

The recovery rate of the samples was calculated with Equation (7) to exclude adsorption, where samples were considered valid if recovery was within 100 ± 5%. HA sample chamber values revealed higher variability than IS and pCS, so sample chamber concentrations were calculated from the buffer chamber concentrations assuming 100% recovery, as buffer chamber values had consistent variability.

(7) % Recovery=mtotal analyzedmtotal applied·100=cbuffer chamber·750 µL+cplasma chamber·500 µLcsample·500 µL·100

### 5.5. Statistical Analysis

GraphPad Prism 10 was used for statistical analysis. In Figure 6, a two-way ANOVA followed by Dunnett’s post hoc multiple-comparisons test was performed and values were considered significant with *p* < 0.05.

## 6. Patents

This study forms part of a wider body of work that has been protected by a patent on “A device for filtering blood, a device for selective heating of blood and a method for controlling a selective heating of blood” (EP 26180095.7; US 19688640). An additional patent was filed but is still pending.

## Figures and Tables

**Table 1:** Binding characteristics of selected prototypical protein-bound uremic toxins.

Uremic Toxin	Free Fraction	Mean Uremic Concentration (µM)	Sudlow Binding Site (Primary/Secondary)	References
Indoxyl Sulfate (IS)	5–10%	150	II/I	[1,9,10,11]
p-Cresyl Sulfate (pCS)	5–10%	200	II/I	[1,9,10,11]
Hippuric Acid (HA)	50–60%	400	Conflicting literature reports	[9,10,12]
CMPF *	<1%	87	I	[1,9,10,11]
Indole-3-Acetic Acid (IAA)	14–16%	12	II/I	[9,11]
Phenylacetic acid (PAA)	24–28%	3.4 × 10^3^	Non-Sudlow site/II	[9,13]

* 3-carboxy-4-methyl-5-propyl-2-furanpropanoic acid.

**Table 2:** Calculated dialyzer clearance (K), reduction ratio (RR) and time-averaged concentration (TAC) in the 5th week of IS, pCS and HA at 37 °C (control) and 43 °C. IS: indoxyl sulfate, pCS: p-cresyl sulfate, HA: hippuric acid.

PBUT	K (mL/min)		%RR		TAC (µM)	
	Control	43 °C	Control	43 °C	Control	43 °C
IS	27.0	31.6	32.5	37.5	68.3	59.9
pCS	21.0	22.4	30.4	32.8	171.3	161.5
HA	132.0	144.3	72.7	75.4	113.3	107.4

**Table 3:** Overview of tested frequency sweep settings.

Frequency Range (MHz)	Frequency Step (MHz)
1–3	0.05
110–112	0.05
1–600	1

**Table 4:** PBUT parameters extracted from Eloot et al. [38]. IS: indoxyl sulfate; pCS: p-cresyl sulfate; HA: hippuric acid; G: toxin generation; K: dialyzer clearance; K_pe_: intercompartment clearance; C_0_: initial concentration.

PBUT	G (mg/min)	K (mL/min)	K_pe_ (mL/min)	C_0_ (µM)	V_plasmatic_ (L)	V_extraplasmatic_ (L)
IS	0.024	27	85	70.82	3.7	12.8
pCS	0.044	21	87	162.59	3.8	6.6
HA	0.136	132	169	134.51	8.4	12.5

## Data Availability

The original contributions presented in this study are included in the article/Supplementary Material. Further inquiries can be directed to the corresponding author(s).

## Supplementary Materials

The following supporting information can be downloaded at: https://www.mdpi.com/article/10.3390/toxins18100443/s1, Table S1: List of blood parameters analyzed in each sample; Figure S1: Temperature effects on blood analytes after a single heating cycle at temperatures between 39 °C and 49 °C; Figure S2: Inter-donor variability in normalized blood analytes across heating cycles.

## Abbreviations

The following abbreviations are used in this manuscript:

ACN	Acetonitrile
BSA	Bovine serum albumin
CKD	Chronic kidney disease
CMPF	3-carboxy-4-methyl-5-propyl-2-furanpropanoic acid
EDTA	Ethylenediaminetetraacetic acid
EM	Electromagnetic
ESKD	End-stage kidney disease
FLR	Fluorescence
HA	Hippuric acid
HD	Hemodialysis
HDF	Hemodiafiltration
HDx	Expanded hemodialysis
HPLC	High-performance liquid chromatography
HSA	Human serum albumin
IAA	Indole-3-acetic acid
ID	Inner diameter
IS	Indoxyl sulfate
KRT	Kidney replacement therapy
LD	Lactate dehydrogenase
MMM	Mixed-matrix membrane
MWCO	Molecular weight cut-off
OD	Outer diameter
PAA	Phenylacetic acid
PBS	Phosphate-buffered saline
PBUT	Protein-bound uremic toxin
PCR	Polymerase chain reaction
pCS	P-Cresyl sulfate
QC	Quality control
RED	Rapid equilibrium dialysis
RF	Radiofrequency
TMP	Transmembrane pressure
UV	Ultraviolet
