# Supplementary material for: Evaluation of Two Non-Invasive Extracorporeal Strategies for Enhancing Protein-Bound Uremic Toxin Removal During Hemodialysis
Source: Toxins (Basel). 2026 Oct 13;18(10):443. doi: 10.3390/toxins18100443 (PMC13709536; doi:10.3390/toxins18100443)
Supplement: Supplementary file 1 [file toxins-18-00443-s001.zip › toxins-4531854-supplementary.pdf]

# Supplementary Materials: Evaluation of Two Non-Invasive Extracorporeal Strategies for Enhancing Protein-Bound Uremic Toxin Removal During Hemodialysis

**João G. Brás, Fokko P. Wieringa, Babette H. Lentferink, Simon Kootwijk, Albert H. Westra, Leonard J. van Schelven, Mies van Steenbergen, Joachim Jankowski, Marianne C. Verhaar, Karin G. F. Gerritsen and Jeroen C. Vollenbroek**

**Table S1.** List of blood parameters analysed in each sample. ALAT: Alanine aminotransferase, LD: lactate dehydrogenase, MCV: mean corpuscular volume, RDW: red blood cell distribution width, MCH: mean corpuscular hemoglobin, MCHC: mean corpuscular hemoglobin concentration, CRP: C-reactive protein, EDTA: ethylenediaminetetraacetic acid, and Li-heparin: lithium heparin.

| Test Name | Marker Type | Anticoagulant |
|---|---|---|
| **ALAT** | Cell damage | Li-heparin |
| **Bilirubin** | Cell damage | Li-heparin |
| **LD** | Cell damage | Li-heparin |
| **Erythrocytes** | General marker | EDTA |
| **Hematocrit** | General marker | EDTA |
| **Leucocytes** | General marker | EDTA |
| **Thrombocytes** | General marker | EDTA |
| **Albumin** | General marker | Li-heparin |
| **Total Protein** | General marker | Li-heparin |
| **MCV** | Hematology | EDTA |
| **RDW** | Hematology | EDTA |
| **MCH** | Hematology | EDTA |
| **MCHC** | Hematology | EDTA |
| **Potassium** | Hemolysis | Li-heparin |
| **CRP** | Inflammation | Li-heparin |

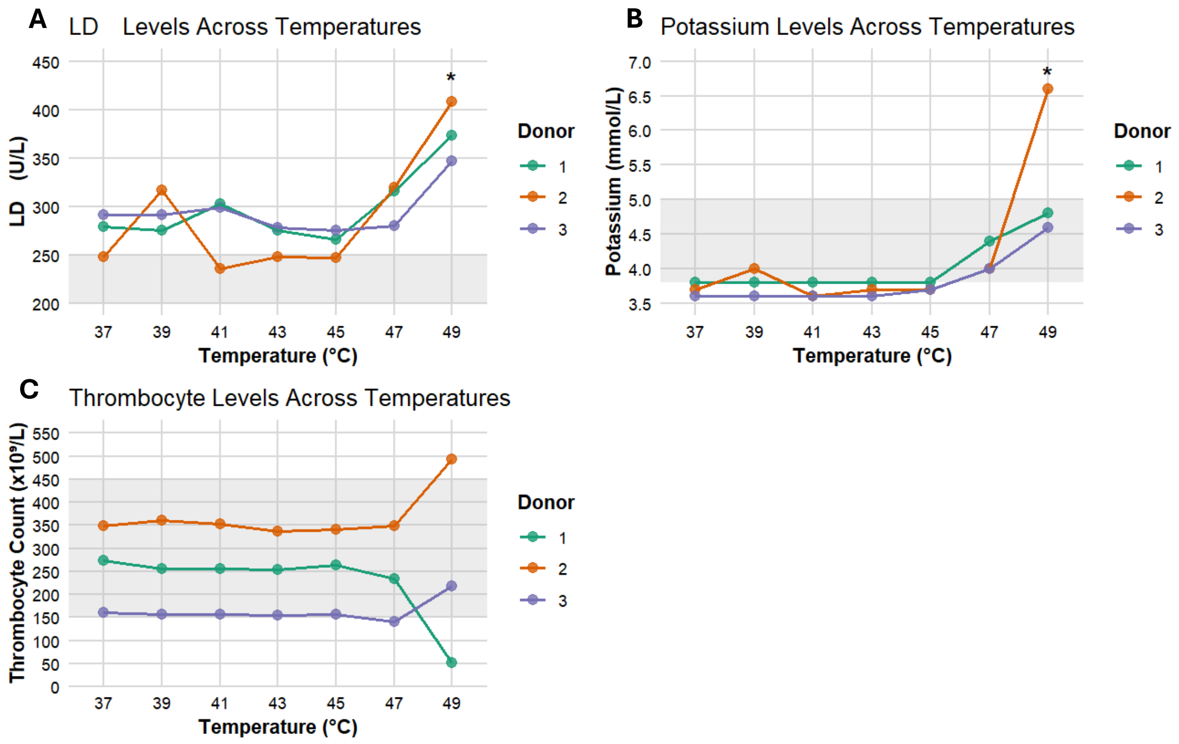


**Figure S1.** Temperature effects on blood analytes after a single heating cycle at temperatures between 39 °C and 49 °C. The 37 °C measurement represents blood that underwent zero heating cycles and serves as the negative control. Grey shaded areas indicate clinical reference ranges: 0 - 250 U/L for LD, 3.8 - 5.0 mmol/L for potassium, and 150 - 450 × $10^9$/L for thrombocytes. * = $p < 0.01$ at 49 °C vs control for LDH and potassium (ANOVA). Thrombocyte levels showed no significant differences between temperatures.

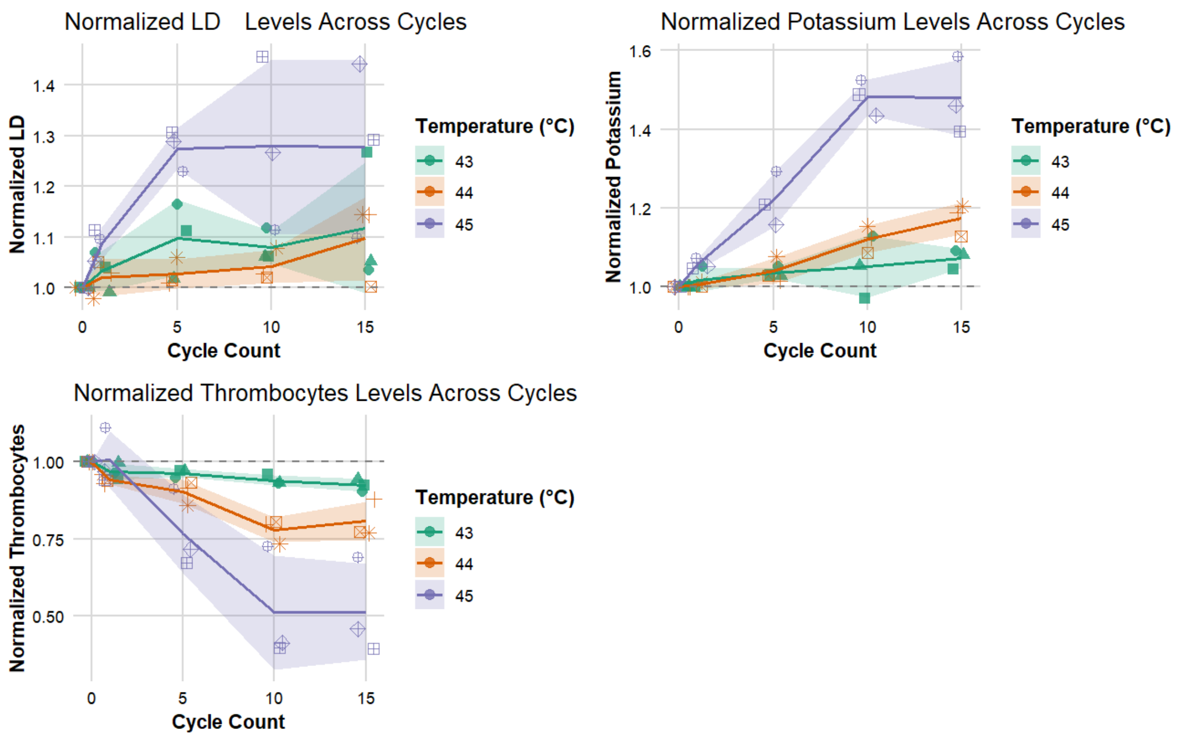


**Figure S2.** Inter-donor variability in normalized blood analytes across heating cycles. Each temperature group consists of measurements from three different donors, with different donors sampled at each temperature, but consistent across all analytes. Data are shown as ratios relative to baseline values at cycle 0 for each donor. Coloured lines connect mean normalized values at each cycle, with shaded ribbons indicating ±1 standard deviation. Jittered points represent individual donor measurements, with different point shapes indicating different donors. The dashed line marks the baseline level (ratio = 1).
